# Action/Verb processing: Debates in neuroimaging and the contribution of studies in patients with Parkinson's disease

**DOI:** 10.1590/S1980-57642014DN81000002

**Published:** 2014

**Authors:** Henrique Salmazo da Silva, Juliana Machado, André Cravo, Maria Alice de Mattos Pimenta Parente, Maria Teresa Carthery-Goulart

**Affiliations:** 1 UFABC – Federal University of ABC. Center of Mathematics, Computation and Cognition. Neuroscience and Cognition Post-graduation. Language and Cognition Research Group (GELC-UFABC), Santo André, SP, Brazil.; 2 Behavioural and Cognitive Neurology Unit, University of São Paulo, School of Medicine, São Paulo, Brazil.

**Keywords:** Parkinson's disease, language disorders, semantics

## Abstract

The objective of the current review was to verify whether studies investigating lexical-semantic difficulties in patients with Parkinson's disease (PD) support the Embodied Cognition model. Under this framework, it is predicted that patients with PD will have more difficulties in the semantic processing of action concepts (action verbs) than of motionless objects. We also verified how and whether these studies are following current debates of Neuroscience, particularly the debate between the Lexical and the Embodied Cognition models. Recent neuroimaging studies on the neural basis of the semantics of verbs were presented, as well as others that focused on the neural processing of verbs in PD. We concluded that few studies suitably verified the Embodied Cognition theory in the context of PD, especially using neuroimaging techniques. These limitations show there is much to investigate on the semantic difficulties with action verbs in these patients, where it is particularly important to control for psycholinguistic variables and the inherent semantic characteristics of verbs in future studies.

## Abstract (pt)

O objetivo deste trabalho foi verificar se estudos sobre dificuldades léxico-semânticas de pacientes com doença de Parkinson (DP) confirmam o modelo da Cognição Incorporada. Este propõe que pacientes com DP terão mais dificuldade no processamento semântico de conceitos de ações (verbos de ação) do que de objetos estáticos. Também verificamos se estes estudos seguem os debates atuais da Neurociência, e especialmente o debate entre o Modelo Lexical e a Cognição Incorporada. Estudos recentes sobre as bases neurais da semântica de verbos coletados através de neuroimagem foram descritos e após, foram apresentados aqueles que focalizaram o processamento neural de verbos em pacientes com DP. Concluímos que ainda existem poucos estudos que verificaram adequadamente a teoria da Cognição Incorporada no contexto da DP, especialmente utilizando neuroimagem. Estas limitações mostram que ainda existe muito para ser investigado sobre dificuldades semânticas de verbos nestes pacientes, principalmente controlando variáveis psicolinguísticas e características semânticas inerentes dos verbos.

## INTRODUCTION

Cognitive Neuropsychology has largely focused on the study of cognitive processes involved in higher order functions, such as language, memory, movement/praxis and so on. During the past 40 years, studies have progressed to also look into the relationship among different functions, a relevant point in current research about the semantics of verbs. The dissociation between verbs and nouns frequently found in studies of patients with cerebral lesions has been criticized by a number of Neuroimaging researchers (for a review, see Vigliocco, Vinson, Druks, Barber & Cappa).^1^ In this confrontation, three different theoretical models were proposed:^2^

[1] a lexical model that argues in favor of a total distinction of the two grammatical classes. In this model, the left temporal lobe underlies the lexicon of nouns, and frontal areas the verb lexicon;
[2] a combinatory model that proposes differentiation restricted to context, but that does not apply to single words. Temporal regions, including the fusiform gyrus, are responsible for the integration of nouns while the left inferior frontal lobe and its medial part are responsible for the integration of verbs;
[3] an emergent model proposing that grammatical classes do not have distinct neural systems. Differences occur due to distinct semantic properties shared by action verbs and events but not by concrete nouns.

The Embodied Cognition theory is one of the subdivisions of the latter approach and assumes that the body plays a fundamental role, being the cause or condition for cognitive development and establishing an interdependency relationship between cognitive processes and the body experiences with the world.^2^ This approach suggests a different view about the acquisition and development of psychological abilities, including motor operations, language and perception, and borrows from neurobiological concepts about neuron connections, in particular the Associative Theory^3^ which postulates that "what fires together, wires together". Focusing on body parts involved in performing particular movements, this approach proposes different networks for mouth, hand and leg movements. Mouth movements are supposed to be represented cortically by more restricted areas in inferior frontotemporal regions; hand movements by inferior and medial frontotemporal areas; and leg movements by superior frontal areas in a more extended way. Thus, according to Embodied Cognition, words that represent actions (verbs) are connected to sensorimotor experiences and the representation is fully integrated to its corresponding action, which means that when a person says "walk" the mental *homunculus* actually "moves". It also proposes an integration of perceptual, attentional, linguistic and motor functions during several activities such as talking about an action, performing the action or simply planning it.^4^

These principles of the Embodied Cognition theory yield a first hypothesis in the Neuroscience field: cognitive tasks involving movement concepts, especially action verbs result in the activation of frontal motor areas. A second hypothesis concerns neuropsychological pathologies: degenerative movement disorders lead to more severe difficulties in action concepts (action verbs) than in motionless objects.

Thus, for the purposes of testing the Embodied Cognition theory and the role of the motor system in Semantics, words need to be classified according to their motor content. For instance, action verbs depict a certain amount of movement (e.g. "to run"), whereas emotional, intellectual or sensory verbs (for example "to please", "to think" and "to see") do not necessarily involve movement. The first group of verbs is also labeled as concrete and the second as abstract verbs. Moreover, nouns have also been analyzed according to movement semantic features. In this sense, the words "car" and "animal" for instance, carry movement semantic information whereas "table" is considered a static/motionless noun. Other features have also been taken into account such as the association between nouns and action verbs (e.g. "a hammer" and "to hammer").

The present review sought to verify how and whether the lexical-semantic studies on lexical-semantic difficulties in patients with Parkinson's disease (PD) are following the current debates in Neuroscience. We start by presenting recent studies on the neural basis of the semantics of verbs followed by investigations about the neural processing of verbs in PD. Lastly, we discuss the state of the art of the two hypotheses mentioned above and outline the investigation possibilities of lexical semantic abilities in these patients.

## NEURAL BASIS OF THE SEMANTIC OF VERBS

Two recent reviews about neuroimaging experiments conducted with cognitively unimpaired adult participants pointed to conflicting results in the ^4^ However, both studies have shown that the tasks requiring greater semantic processing amplify the differences between two grammatical classes -nouns and verbs, and that there is a trend of more activation areas for verbs when compared to nouns, suggesting that the former have a higher semantic complexity.^4^

EEG studies, due to their temporal precision, have shown an anticipation of electrophysiological registrations in tasks using verbs when compared to tasks that used nouns. Activations at around 350-450 milliseconds (N400 effect, considered a signal of semantic processing) showed a similar pattern between verbs and nouns.^5^ ERP evidence showing an N400 effect on the right hemisphere and early activation of motor areas for action word processing, corroborated the Embodied Cognition theory and indicated a somatotopic organization of language.^6^ Confirming the early activation in verb processing, a comparative study in children aged 8 or 9 and adults found similar N400 effects in both groups, but pointed to a difference in N300 effects.^7^ Both N300 and N400 components seem to be sensitive to semantic properties of the presented stimuli. However, while the N400 has been found with the use of different kinds of stimuli (such as words that violate the semantic context of a sentence, or words not semantically related to a previous list of words, or even pictures that are not related to an olfactory prime), the N300 appears to be specific to semantic incongruence between a word and a subsequent picture. Given that there was a difference in N300 only for objects, the authors of the study suggested that action verb representations continue to solidify through middle childhood. Noteworthy differences between action verbs and visual nouns (not related to movements) were found at around 120-220 milliseconds after stimulus presentation, but no differences were observed between verbs and nouns referring to actions.^8^

Studies that took into consideration movements involving different body parts have demonstrated that differences among verbs performed with different body parts are evident around 250 milliseconds after stimuli presentation.^3^ In spite of the low spatial resolution of electrophysiological data, legs and mouth movement verbs confirmed the associative theory model yet hand verbs did not. Frequency and familiarity, factors that can influence the speed of lexical access and semantic processing, have been controlled in several studies, but one of the reasons for the absence of coherence between hand/arm verbs and their respective motor areas could be that numerous stimuli are needed for an electroencephalography (EEG) experiment, and in some studies, semantic criteria for hand verbs were not fully satisfactory. Hand/arm verbs have many semantic variations, such as verbs of change of state (E.g. "to cut") and verbs that need tools (E.g. "to hammer") among others, and these peculiarities were not always taken into account. Another possibility could be the lack of differentiation of the specificity levels of action verbs, evidenced during acquisition^10^ and during linguistic degeneration processes.^11^ In the example above, "to cut" is a generic verb, since there are many forms of cutting, and "to saw" is a specific verb, since there is only one way of doing this: with a saw in a specific manner. Specificity criteria do not overlap with the distinction between manner/instrument verbs; since several actions that do not require instruments can also be specific (such as "to chew").

Magnetoencephalography (MEG) studies about the lexical semantic properties of verbs remain scarce. However, due to their temporal and spatial precision, they were able to confirm the early activation of action concepts and suggested better results regarding location. Semantic category distinctions were found around 150 milliseconds after stimulus onset, with action words activating frontocentral motor areas more strongly whereas more visual words (not related to any movement) activated the occipitotemporal cortex, confirming the sensorimotor activation for action verbs.^12^ When comparing the processing of verbs that involve different body parts in a lexical semantic retrieval task and the proper movement of that part, the Embodied Cognition theory was also confirmed, with a correlation found between the verbs and the actual movements.^13^ Therefore, the few MEG studies included in the current review were in accordance with the Embodied Cognition theory. Perhaps, as this technique allows the use of fewer stimuli to detect activation, these studies were able to control the semantic variables and the use of prototypical verbs.

On the other hand, fMRI studies have shown divergent results. The counterpoint between the Embodied Cognition and the lexical model has been the most frequent topic of discussion. Based on Pulvermüller's associative theory, as expected, representations involving mouth actions activated the inferior prefrontal gyrus.^14,19^ When investigating leg movements, the majority of findings pointed to prefrontal and superior frontal activations, coinciding with the *homunculus* motor representation, despite medial prefrontal activation observed for pressure movements of the legs.^20^ These studies showed that the representation of hand movements and leg movements overlap, incongruent with the theoretical model. However, when there was a semantic distinction between "to hit" and "to cut" (both hand related) verbs, the former activated superior motor areas and the latter medial premotor areas.^19^ According to the authors, the involvement of the premotor area is justified by a higher degree of planning, since the majority of "cutting" verbs need tools. Moreover, body action verbs ("to run") have been shown to depend upon the motor and premotor cortex; face movements, including speaking, upon posterolateral temporal cortex; change of state ("to crush") verbs upon ventral cortex, and use of tools ("to dig") on the frontoparietal and temporal network.

The role of the posterior-lateral-temporal cortices (PLTC) was also reported in comprehension of action words when compared with comprehension of nouns.^19^ This activation was explained by a network in which the PLTC is connected to the middle temporal area - which processes visual motion - and to the right superior temporal sulcus, which is important for biological motion perception. From this point of view, the PLTC is important for verb processing because the comprehension of action concepts requires visual-motion representations.

On the other hand, based on the lexical model, studies have shown that PLTC are considered regions where all grammatical classes can be recruited, and thus their activation reflects the retrieval of modality-independent representations of event concepts, including nouns and verbs.^20^ This position was confirmed in an fMRI experiment after semantic-relatedness judgments on word pairs with different amounts of visual-motion information. After these judgments, the stimuli were divided into high-motion words (which included action verbs and nouns representing animals), and low-motion words (that included verbs referring to mental activities and nouns representing inanimate natural items). Whole-brain analyses showed that no region was more active for high-motion compared to low-motion words at the corrected threshold. Moreover, random effects analyses replicated greater activity for action verbs than names of animals in the PLTC. The authors concluded that all concepts were abstracted at posterior parts of the brain and were independent of the degree of movement.

Finally, supporting the lexical theory, fMRI experiments have compared congenitally blind individuals and controls in a word processing task. Controls showed greater activation of the left medial temporal gyrus.^21^ Congenitally blind subjects had similar activation patterns in a semantic judgment task of action verbs. According to this group of authors, lexical semantic knowledge is independent of sensorimotor experience and is organized according to conceptual properties. However, researchers form the same group,^22^ who had late-blind and congenitally blind participants perform a task of tool-size evaluation, observed specificity in blood-oxygen-level-dependent responses for tools in the left inferior parietal lobule and the left anterior intraparietal sulcus in the blind group. This result was interpreted as a possibility that sensorimotor processes are responsible for tool representation specificity in parietal cortex areas.

Therefore, since the methodological criticism of Vigliocco et al.^2^ and Crepaldi et al.,^4^ there has been stricter control on stimulus choice in fMRI studies and control over the kind of semantic processing demanded by the task. For instance, differences in deeper processing were shown by studies using naming picture tasks that produced higher activation in more extended areas,^7,23^ whereas verbs compared to nouns in morphologic cueing tasks ("to+verb" or "the+noun") strongly activated the medial temporal gyrus and left superior temporal gyrus.^24^ Nevertheless, the results of fMRI studies are still conflicting and their interpretation reflects the complexity of the semantic of verbs and the underlying theoretical approaches.

Research using Transmagnetic Stimulation techniques (TMS) have shown the involvement of the frontal cortex, more specifically the medial frontal gyrus and the inferior frontal gyrus, in the processing of action words:^1^

[1] slower latency times have been found for the production of verbs and pseudoverbs (which is a pseudoword with the morphological structure of a verb, for instance "he wugs") when compared to nouns after prefrontal cortex suppression;
[2] the use of repetitive TMS in the left prefrontal cortex (medial frontal gyrus) pointed to a specific interference in motor cortex in the processing of verbs and action nouns when compared to abstract verbs and nouns regarding objects; and
[3] the stimulation in primary motor cortex promoted facilitation effects in recognizing action words at around 500 milliseconds.

Another study, through inhibition of left primary motor cortex related to hand area stimulation, promoted a greater inhibition of concrete verbs compared to abstract verbs. This effect showed the involvement of the motor cortex in action verb processing.^25^ However, another study using the same procedures showed that motor stimulation increased the motor evoked potentials only when TMS was applied 300 milliseconds after an action-related verb. However, the results also suggested that with repetition, the primary motor cortex was no longer necessary for these verbs.^26^ Moreover, also using TMS techniques, participation of the PLTC was found for action and abstract verbs as well in semantic analyses of both verbs and nouns.^27^

A recent meta-analysis,^28^ however, which sought to verify the embodied cognition theory consistency in TMS studies, showed significant agreement between brain regions within or adjacent to visual motion areas, but no consistency was found in motor or premotor cortices.^28^

To sum up, controversies between lexical and embodied accounts continue but have promoted a high output of neuroimaging studies. These studies have discussed grammatical differences and sensorial-motor area participation in verb processing and also contributed to the understanding of verb processing and its semantic organization. The earlier neurophysiological activation for action verbs found in adults but not in children confirms the semantic complexity of the verbs, and studies that took into account the different semantic classes of verbs resulted in a better understanding of verb processing. These advances have contributed to the diagnosis and cognitive intervention of patients with verb processing difficulties.

## VERBS AND ACTIONS IN THE BRAIN: INSIGHTS FROM PD

Pathologies affecting primarily the motor system constitute an interesting model to investigate the role of motor areas in the processing of action verbs. The greatest number of studies in this field has been undertaken on patients with PD, although some important contributions have come from studies conducted on other movement disorders.^29,30^

PD is a neurodegenerative disease characterized by bradykinesia (slowness of movement), rigidity, tremor, gait and posture problems. It is caused by a progressive loss of dopamine in the nigrostriatal tract, reducing the projections of the basal ganglia to the frontal motor regions.^31^ The deficits in the dopaminergic pathways cause hypo-activation of the supplementary motor area and primary motor cortex, and hyper-activation of the ventral premotor cortex, reflecting a compensatory mechanism.^31,32^ PD is an interesting framework for investigations into the semantics of verbs due to the possibility of modulating motor deficits by exploring the effects of medication (patients ON and OFF Levodopa) and also of surgical interventions in the performance of patients. In this section we will summarize the findings in this area and discuss their contributions to support the Embodied Cognition theory.

As mentioned previously, semantic deficits have been more extensively studied in PD than in other diseases predominantly affecting the motor system. However, the literature in this field is still scarce. A Pubmed and Scopus search conducted in February/2014 using the terms "action verb" OR "verb" OR "verbs" AND "Parkinson's disease" with no time restriction retrieved only 30 studies. After excluding reviews and studies not related to action/verb semantics in PD, the number of manuscripts totaled 18 studies. Table 1 summarizes the methodologies and main findings of these studies analyzed in the present review to determine their contributions to the debate on how action/verb semantics is represented in the brain, particularly with regard to the Embodied Cognition versus lexical/grammatical class theories.

**Table 1:** Summary of studies investigating action/verb semantics in patients with Parkinson's disease.

Behavioral evidence					
Authors	Participants	Medication	Tasks	Experimental details	Principal findings
Bertella et al. (2002)^50^	22 PD patients and 20 healthy controls.	Not specified	52 pictures of objects and 50 pictures of actions to be orally named.	Not specified.	A verb/noun dissociation with a relative deficit for verbs was found in PD patients.
Péran et al. (2003)^51^	34 nondemented PD patients and 34 healthy controls.	ON State	Noun- and verb-generation tasks, using two intracategory (noun/noun and verb/verb generation) and two intercategory (noun/verb and verb/noun) tasks.	40 concrete nouns and 40 action verbs matched for lexical frequency and length.	PD patients were impaired on the tasks involving verb/verb and noun/verb generation. Significant correlations were found between DRS scores and performance on the noun/verb task.
Cotelli et al. (2007)^34^	32 PD patients in initial stage (ON) and 15 healthy controls.	ON State (32)	Object Picture naming (60) and verbs with hands (60), of varying difficulty.	Objects and verbs were matched by word frequency and word length.	PD patients had impairments in naming objects and actions, with poorer performance for actions.
Crescentini et al. (2008)^48^	20 PD patients in initial stage and 20 healthy controls.	ON State (16)	Verbs and names generation (name-verb, name-name).	Concreteness and frequency were calculated.	PD patients had deficits both for verbs and names generations, with poorer performance for verbs.
Castner et al. (2008)^52^	8 PD patients who had received surgery for deep brain stimulation (DBS) and 15 healthy controls .	With and without DBS	4 probe-response conditions-namely, noun-noun, verb-noun, noun-verb and verb-verb conditions.	Nouns and verbs were matched for spoken lexical frequency.	Without DBS, patients had a selective deficit in verb generation compared to the control group. DBS resulted in more errors in patients for the noun-noun and verb-verb conditions. Verb generation errors were correlated with item selection constraints (i.e. the degree to which a response competes with other response alternatives) in the DBS, but not without stimulation.
Boulenger et al. (2008)^41^	10 PD patients without dementia (ON and OFF) and 10 healthy controls.	ON and OFF States	Priming task- 140 words (70 action verbs with hands and 70 concrete names).- 140 pseudowords (70 pseudoverbs and 70 pseudonames).- 280 non-words.	Verbs and nouns used in the task were matched for frequency, length, bigram and trigram frequency and phonological complexity Imageability and age of acquisition were also taken into account.	Priming effect for verbs was smaller in OFF than in ON situation. Reaction times modulated by Levodopa.
Rodríguez-Ferreiro et al. (2009)^53^	28 PD patients without dementia.28 patients with AD.28 healthy controls.	Not specified	Naming 50 object pictures and 50 action pictures.Pictures of verbs and naming were extracted from Snodgrass and Vanderwart.	These were matched by name agreement and frequency, age of acquisition, imageability and number of phonemesVisual complexity was higher in verb pictures.	Naming actions was more difficult for PD patients.
Silveri et al. (2012)^35^	12 PD patients without dementia.	ON and OFF states	Naming 50 object pictures and 50 action pictures.	Stimulus were matched by word frequency, age of acquisition, typicality and agreement of response; not matched by imageability (lower for verbs) and word length.	OFF resulted in less accuracy and greater reaction time, especially for verbs.
Herreira and Cuetos (2012)^39^	20 PD patients.20 healthy controls.	ON and OFF states	Semantic word generation of 10 words and 10 verbs.	Without restriction of verbs generation.	OFF was associated with the generation of less semantically similar verbs than ON. Generation differences in verbs was evident when PD OFF were compared with controls.
Herreira et al. (2012)^49^	22 PD patients.20 healthy controls.	ON and OFF states	4 verbal fluency tasks: phonological fluency, semantic fluency (animals and supermarket) and action fluency (things you can do).	Patients were asked to produce as many words as they could from each category in 60 s.	Differences in ON and OFF were found in number of words. PD OFF produced fewer words on the phonological and action fluency tasks compared to controls. Differences were found between PD OFF and controls in frequency for the action-word category.
Herreira et al.(2012)^44^	49 PD patients and 19 healthy older adult controls.	ON State	Participants were asked to name pictures of Actions, extracted from the Object and Action naming battery or the IPNP- International Picture Naming Project database.	Subsets of 25 high (e.g. “to dig”) and low (e.g. “to sleep”) motor-association action verb were matched for visual complexity, name agreement, frequency, age of acquisition, imageability and number of syllables and phonemes.	PD patients obtained poorer results in response to pictures with high motor content compared to those with low motor association. PD patients performance appeared to be negatively affected by the level of motion-related semantic content associated to each verb.
Ibáñez et al. (2012)^42^	17 PD patients, 15 healthy controls, 2 epilepsy patients undergoing surgery.	ON State	Action and sentence compatibility effect and Kissing and dancing test.	Judgments of actions with and/or without hands, psycholinguistic variables not specified.	PD patients with better cognitive performance had fewer deficits on both tests.
Fernandino et al. (2013)^43^	20 PD patients without dementia and 20 healthy controls.	ON State - Levodopa (17)	Priming TasksLexical decision - 80 verbs (action and abstract verbs) and 80 pseudowords).Semantic Judgment - 120 action verbs and 120 abstract verbs.	Stimuli were matched in number of letters, phonemes, syllables, orthographic and phonological neighbors; lemma frequency.	PD patients had higher reaction times to verbs (equal for action and abstract verbs) in Lexical Decision and were less accurate for action verbs than abstract verbs in semantic judgment.Differences in Priming effect were not observed in abstract and action verbs.
Fernandino et al. (2013)^40^	20 PD patients without dementia and 20 healthy controls.	ON State - Levodopa (17)	Judgment of sentences containing action verbs used with literal and figurative meanings (idioms), control conditions with other abstract sentences.	Conditions were matched for sentence length (letters, phonemes, syllables, and words), response times and accuracy in lexical decision for the content words in the sentence, according to the English Lexicon Project database.	Compared to controls, PD patients had higher reaction times for action verbs both in literal and figurative sentences.
Kemmerer et al. (2013)^32^	10 PD patients without dementia and 10 healthy controls.	ON and OFF states	Similarity judgment of four classes of action verbs (running, hitting, cutting, speaking) and two classes of non-action verbs (change of state, emotional states).	Verbs were similar in frequency and letter length. Psych verbs (emotional states) had more letters than others.	Patients and controls had similar accuracy rates but higher reaction times under all conditions.
**Neuroimaging studies**					
**Authors**	**Participants**	**Medication**	**Tasks**	**Experimental details**	**Principal findings**
Péran et al. (2013)^37^	8 PD patients without dementia].fMRI study.	ON and OFF states	Exhibition of pictures of biological objects and tools to: 1. name objects (objn) 2. Naming of action that could result from the figure (Gena) 3. creating objects for action (MSoA).	Pictures were extracted from Snodgrass and Vanderwart normalized data set, and balanced for frequency and length.	Compared to objn: Gena generated greater activation of the left prefrontal cortex and left medial precuneus; - MSoA generated greater activation of prefrontal cortex and occipital-parietal junction bilaterally. State ON> OFF: (Gena) - premotor areas (MsoA) - premotor areas and thalamus.
Peran et al. (2009)^38^	14 PD patients without dementia in ON state.fMRI study.	ON state - PD Medications	Pictures of biological objects and tools Exhibition whose interest was: 1. naming objects; 2. election of action that could result from picture.	Pictures were extracted from Snodgrass and Vanderwart normalized data set, and were balanced for frequency and length.	Preferential involvement of the prefrontal cortex, Broca's area and anterior cingulate cortex to generate verbs. There was no specific activation of objects and verbs as the category.
Letter et al. (2012)^47^	7 PD patients.EEG - LORETA.	ON and OFF States	Reading of 30 action verbs and 30 non-action verbs.	The series consisted of 30 hand action verbs (e.g. to sew, to point) and 30 non-action verbs (e.g. to leave, to develop). All verbs consisted of two syllables, matched with respect to word form frequency, and imageability.	ON state resulted in strong currents to all cerebral areas analyzed, with most obvious difference for ON state in the left hemisphere.

PD: Parkinson's disease; DBS: Deep Brain Stimulation; AD: Alzheimer's disease; ON state: cognitive evaluation was undertaken in a patient under the influence of medication; OFF state: cognitive evaluation was undertaken in a patient not under the influence of medication; EEG: Electroencephalography; Loretta: Low Resolution Electromagnetic Tomography.

Apparently not all of these studies were methodologically designed to test the Embodied Cognition assumptions and this represents a major limitation for the current review. However, results are often explained/discussed as supportive of this theory and it is not always clear why alternative explanations were not considered. A common finding of the studies reviewed here is that PD affects verb processing and therefore frontal cortical-subcortical circuits and structures are engaged in action/verb processing. The deficits are more intense in the absence of L-dopa (OFF state), predisposing patients with PD to longer reaction times in naming tasks, decisions and semantic judgments of action verbs compared to patients under the influence of medication (ON state) and to healthy controls matched for age, sex, and education.^33-35^ Levodopa is believed to play an important role in restoring activity of the motor circuitry involved in the semantic processing of actions/verbs.^33,34^ The extent and nature of the contribution of these areas to action/verb semantic processing however, is less clear in the literature. Some issues of intense debate in cognitive neuroscience and cognitive neuropsychology related to this topic are discussed below.

The first question in the debate is: Do patients with motor disorders have impairment in the processing of action semantics or are their difficulties due to a problem restricted to the grammatical class of verbs? To demonstrate that the difficulty involves "action semantics" and not purely "verbs", studies need to demonstrate that other types of verbs are unimpaired. Dissociation between action and non-action verbs would favor the Embodied Cognition claim whereas verb/noun dissociations, although elucidating, cannot rule out the lexical hypothesis.^36^

Comparison between verbs and nouns was used in the 15 studies reviewed and all of them yielded behavioral evidence of a disproportionate deficit for verbs compared to nouns. A host of different tasks have been employed, such as naming of action verbs,^34,35,37,38^ generation of semantically similar verbs,^39^ judgments of semantic similarity^32^ and of literal and figurative sentences involving body action verbs,^40^ identification of action verbs^41^ and the interaction between contextual understanding of action verbs and motor responses.^42^

However, bar a few exceptions,^32,40,43,44^ these studies did not investigate processing differences between distinct types of verbs (action, non-action, emotional and abstract verbs) while some did not control for many psycholinguistic variables, such as length, frequency, imageability, age of acquisition, visual complexity, among others. Another limitation is the absence of healthy control groups.^35^ This is an important issue, considering that verbs are more demanding of cognitive resources (for a review see Matzig et al.).^45^

A second issue of discussion in the literature concerns the studies that employed an approach which is methodologically appropriate to address the Embodied Cognition theory. In this case, are results interpreted in terms of a relationship or a causal role between action semantics and frontal circuits? If the integrity of the motor system is necessary for action semantics a clear impairment must be demonstrated in PD. However, if the motor system contributes, but is not necessary for action verb processing, then patients may present no clear deficits when compared to controls, or exhibit time but not accuracy differences.^32^ The latter would not refute the Embodied Cognition claim but would challenge the strong form of this theory. Controversies persist in this area with some researchers favoring the strong version of the theory,^40,43^ stating that integrity of the motor system is necessary to process action verbs even when used with figurative meaning whilst others support (albeit with some reservations) the weak version of embodied cognition (motor representations may enrich the semantics of verbs but are not necessary for the comprehension/production of verbs).^32^ However, Kemmerer et al.^32^ mentioned that if they had considered only one type of action verb (the category "cutting") and ignored the other action verbs addressed in their study, the findings would support the Embodied Cognition theory in the sense that patients were less accurate on these verbs compared to controls. The results of this study raises the question of whether specific (verbs with precise and detailed kinetic movements and that usually require a tool)^46^ vs. general verbs have different neural representations and whether this question could help clarify PD action/verb deficits and cast light on issues regarding the Embodied Cognition theory.

Moreover, if the Embodied Cognition theory is correct, neuroimaging studies with PD should point to somatotopic organization of action verbs related to specific body parts or different patterns of activation for action verbs compared to other verbs. However, no studies addressing the question in this population are available. Only two studies used fMRI techniques to study action verb processing in PD.^37,38^ Their results confirmed the hypothesis of a relationship between motor striatofrontal dysfunction and impairment of verb processing, either due to higher level of difficulty with action verbs compared to nouns^37^ or to the observation of a relationship between increased activation during a verb generation task and increased motor-frontal dysfunction in different brain regions.^38^ One study employed EEG techniques^47^ and suggested that dopamine (patients in ON state) had elevated differences in coherent neural activity when processing action compared to non-action verbs. Taken together, the findings with neuroimaging techniques are difficult to relate directly to the Embodied Cognition Theory.

Finally, a common challenge in these types of studies is to address the influence of psycholinguistic variables known to have an impact on language performance such as frequency, familiarity, imageability, extension, visual complexity and age of acquisition, when comparing different categories of verbs. If these factors, or at least most of them, are not taken into account then differences between verbs cannot be explained without limitations. Studies often report frequency and extension but overlook other aspects.

In summary, few studies were appropriate to verify the Embodied Cognition theory in the context of PD, especially using neuroimaging techniques. These limitations show that there is much to investigate on the semantic representation of action verbs. Considering the hypotheses mentioned in the introduction, the studies conducted so far have been able to show a disadvantage for verbs compared to motionless objects (nouns) in PD. However, evidence of a causal relationship between motor processing and action semantics can be derived from only two studies^40,43^ and requires further investigation.

## CONCLUSIONS AND FINAL CONSIDERATIONS

Our review about neural representations in verb processing has shown that:

[1] the debate between Embodied Cognition and Lexical models continues. It has resulted in a great number of neuroimaging studies and their results are interpreted following the authors' positions in most of the experiments;
[2] control of stimuli and of the cognitive processing elicited by the tasks has been improving over recent years, but in general, tasks requiring more semantic processing amplify the differences between the two grammatical classes (nouns and verbs), resulting in greater activation in more areas for verbs compared to nouns, consistent with the higher semantic complexity of verbs;
[3] when the researchers focused on the body parts performing the movement, similar neurophysiological activation with the counterpart motor representation was readily found for leg and mouth actions, but not for arm actions. Hand/Arm movements in human beings comprise a large variety of actions, thus semantic classification needs to be taken into account, such as the use of instruments and the degree of specificity.

Based on these findings, we examined research into verb processing of PD patients. Studies are recent and scarce. We noted that:

[1] difficulties in patients with PD did not show a clear advantage for any one model of semantic representation;
[2] neuroimaging studies with PD patients are rare, and only one had verified the effects of deep brain stimulation in patients with PD aiming at studying the possible contribution of the subthalamic nucleus connections to verb semantics;
[3] most of the research focused on the greater difficulty for verbs compared to nouns, but only a few investigated processing differences between distinct types of verbs.

Finally, some questions remain open in the literature, such as:

[1] the possible differences in neural representation of verbs of different parts of the body;
[2] the processing of action verbs in literal and figurative language;^40^
[3] comparisons between action and non-action verbs, specific and generic verbs and verbs which evoke emotional responses ("to feel lonely", "to love"), visual processing ("to color", "to dye") and others ("to rain");
[4] associations between the degree of severity of PD and the loss of semantic characteristics of the verbs, such as specificity, concreteness, force of movement, etc.; and
[5] The impact of executive functions and working memory on motor representation of semantics. With regard to this last question, dissociations between verbs and objects seem to be related with poorer performance on tasks of visuospatial and verbal memory^34^ as well as executive functions.^48^

In sum, much more work must be carried out to understand how the brain represents the complex semantics of verbs and to ascertain which semantic strategies can help patients with specific difficulties in verb processing.
